# Selenium-incorporated mesoporous silica nanoparticles for osteosarcoma therapy†

**DOI:** 10.1039/d2bm02102a

**Published:** 2023-04-19

**Authors:** Lei He, Pamela Habibovic, Sabine van Rijt

**Affiliations:** a Department of Instructive Biomaterials Engineering, MERLN Institute for Technology Inspired Regenerative Medicine, Maastricht University P.O. Box 616 6200 MD Maastricht The Netherlands s.vanrijt@maastrichtuniversity.nl

## Abstract

Selenium (Se) compounds are promising chemotherapeutics due to their ability to inhibit cancer cell activity *via* the generation of reactive oxygen species (ROS). However, to circumvent adverse effects on bone healthy cells, new methods are needed to allow intracellular Se delivery. Mesoporous silica nanoparticles (MSNs) are promising carriers for therapeutic ion delivery due to their biocompability, rapid uptake *via* endocytosis, and ability to efficiently incorporate ions within their tunable structure. With the aim of selectively inhibiting cancer cells, here we developed three types of MSNs and investigated their ability to deliver Se. Specifically, MSNs containing SeO_3_^2−^ loaded on the surface and in the pores (MSN-Se_L_), SeO_3_^2−^ doped in the silica matrix (Se-MSNs) and Se nanoparticles (SeNP) coated with mesoporous silica (SeNP-MSNs), were successfully synthesized. All synthesized nanoparticles were stable in neutral conditions but showed rapid Se release in the presence of glutathione (GSH) and nicotinamide adenine dinucleotide phosphate (NADPH). Furthermore, all nanoparticles were cytotoxic towards SaoS-2 cells and showed significantly lower toxicity towards healthy osteoblasts, where Se doped MSNs showed lowest toxicity towards osteoblasts. We further show that the nanoparticles could induce ROS and cell apoptosis. Here we demonstrate MSNs as promising Se delivery carriers for osteosarcoma (OS) therapy.

## Abstract 2

Se-incorporated MSNs were synthesized by loading SeO_3_^2−^, doping SeO_3_^2−^, and incorporating SeNP into MSNs. Se-incorporated MSNs showed GSH/NADPH sensitive Se release, selective OS cell inhibition and ROS induction.

## Graphical abstract

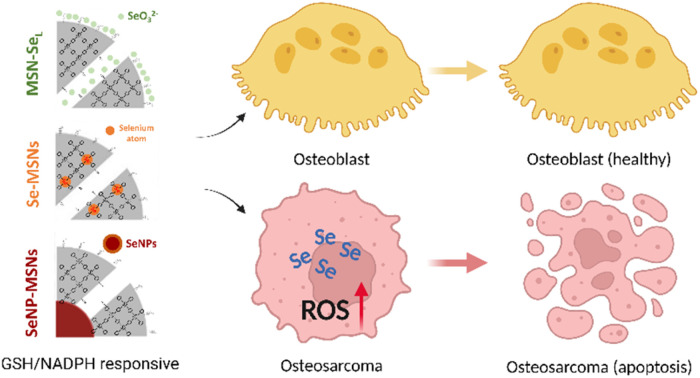

## 1. Introduction

Osteosarcoma (OS), one of the most common primary malignant tumors originating from bone tissue, is usually observed in adolescents and in the elderly population (American Cancer Society).^1^ OS can result from predisposing factors including bone diseases (Paget's disease), radiation exposure, and heritable genetic mutation (*e.g.*, Li-Fraumeni syndrome germline mutation).^2^ OS frequently occurs in the long bones, close to the joint, such as the femur (42%), tibia (19%) and humerus (10%) and is less prevalent in the skull, jaw, pelvis, ribs and spine.^3^ Even though OS is considered a relatively rare disease compared to other cancers, its incidence has been increasing over decades.^4^ Patients diagnosed with OS often suffer from local pain and are at high risks of metastasis and recurrence, highly affecting the patient's quality of life.^5,6^ Gold standard clinical treatment of OS consists of local surgery combined with adjuvant/neoadjvant chemo-radiotherapy.^7^ However, the efficacy of current chemo-radiotherapy treatments has declined due to drug resistance. Moreover, severe side effects and low tumor selectivity are other significant problems associated with current treatments.^8^ These issues have resulted in plateauing of survival rates of OS patients. Specifically, patients with localized (non-metastatic) OS have 5-year survival rates of approximate 60–65%, a percentage that has remained constant over decades. Moreover, patients suffering from systematic recurrent and metastatic OS have 5/10-year survival rates have been constantly as low as 30%.^9^

Research into alternative treatment methods has focused on developing new types of chemotherapeutics (*e.g.*, based on organic drugs, microRNA, and ions), photodynamic therapy (*e.g.*, using graphene oxide), hypothermia (*e.g.*, using Fe_3_O_4_ nanoparticles) and immunotherapy (*e.g.*, using vaccines).^10–13^ Among these new therapies, bioinorganics, including ions and inorganic nanoparticles based on selenium (Se), zinc (Zn) and iron (Fe), represent interesting candidates for OS therapy, because they show unique anti-OS activity. For example, inorganic Se can induce programmed cell death by generating reactive oxygen species (ROS).^14^ Promoting death in OS cells through ROS has been identified as an interesting pathway as endogenous ROS levels in OS cells are higher than in healthy cells and can lead to the selective inhibition of OS cells.^15^

For Se to be effective, it needs to be administered at relatively high doses in order to behave as a pro-oxidant. Indeed, when present at low doses, Se can maintain metabolism and repair DNA of cells.^16^ Moreover, obtaining the required intracellular Se levels is difficult as Se cell internalization *via* ion channels varies from cell to cell, and Se accumulation in the microenvironment can lead to harmful side-effects.^17,18^ Nanoparticle (NP)-based drug delivery systems can be used to circumvent these issues by providing higher control over intracellular Se delivery. In this regard, mesoporous silica NPs (MSNs) are promising because they are efficiently transported into cells *via* endocytosis, and ions can easily be incorporated in their tunable mesoporous structure. For example, we have recently shown that MSNs can be modified for multiple ion delivery by incorporating ions in the matrix and mesopores and that the mode of ion incorporation strongly affect the overall bioactivity of the NP.^19,20^ Moreover, MSNs can be surface modified to allow controlled ion release by using specific stimuli, such as pH, to prevent unwanted cargo release, limiting harmful side-reactions.^21^ Finally, MSNs have intrinsic bone regenerative capabilities, which may be beneficial for use in OS patients to regenerate dissected bone containing tumor.^22^

**Scheme 1:**
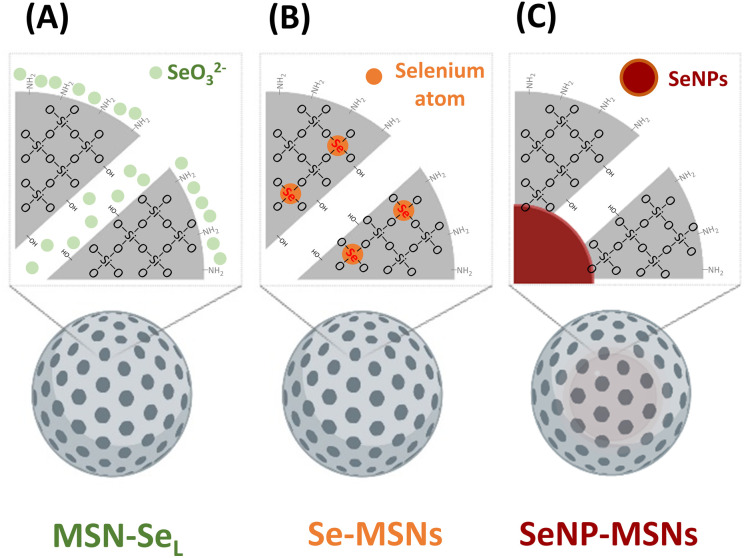
Se-incorporated MSNs synthesized in this study. (A) MSNs with SeO_3_^2−^ loaded in the pores and on the surface (MSN-Se_L_); (B) MSNs with SeO_3_^2−^ doped in the network (Se-MSNs); (C) SeNPs incorporated MSNs core–shell structure (SeNP-MSNs).

Although several reports have shown that MSNs can be doped with therapeutic ions such as Cu^2+^, Sr^2+^, and Fe^3+^,^23–25^ there are no studies reporting on doping Se into MSNs. Several studies have reported on coating Se nanoparticles (SeNPs) with mesoporous silica.^26–28^ In this study, we aimed to incorporate Se into MSNs using different incorporation modes and investigate their *in vitro* bioactivity (Scheme 1). Three modes of Se incorporation were investigated: in the first group, SeO_3_^2−^ (Se^4+^) was directly loaded into the mesopores and on the surface of amino functionalized MSNs (MSN-Se_L_). In the second group, SeO_3_^2−^ was doped into MSNs inorganic framework *via* ion substitution (Se-MSNs). In the third group, elementary Se NPs (SeNPs) were coated with mesoporous silica to create a core/shell structure (SeNP-MSNs). We report Se-incorporated MSNs synthesis and characterization, degradation and ion release rates as well as their cytotoxicity in OS cells and normal osteoblasts and ability to induce ROS and apoptosis.

## 2. Materials and methods

### 2.1 Chemicals

Tetraethyl orthosilicate (TEOS), 3-mercaptopropyl triethylsilane (MPTES), triethanolamine (TEA), 3-aminopropyl triethoxysilane (APTES), cetyltrimethylammonium chloride (CTAC), cetyltrimethylammonium bromide (CTAB), bis[3-(triethoxysilyl) propyl] tetrasulfide (BTES), ammonium fluoride (NH_4_F), hydrochloric acid (HCl, 37%), ammonium nitrate (NH_4_NO_3_), sodium selenite (Na_2_SeO_3_), l-ascorbic acid (*V*_c_), bis[3-(triethoxysilyl)propyl] tetrasulfide, and ATTO 488-maleimide dye, sodium cacodylate trihydrate ((CH_3_)_2_AsO_2_Na·3H_2_O), G418 disulfate salt (C_20_H_40_N_4_O_10_·2H_2_SO_4_) were purchased from Sigma Aldrich GmbH (Germany). Absolute ethanol was obtained from VWR (US). Saos-2 cells and hFOB 1.19 cells were purchased from ATCC (US). CellTiter 96® AQ_ueous_ non-radioactive cell proliferation assay (MTS) was bought from Promega (US). DCFDA/H_2_DCFDA (cellular ROS) assay kit was purchased from Abcam (UK). Apoptosis assay (Annexin V/Propidium Iodide (PI)) was bought from Thermo Fisher Scientific (US).

### 2.2 Preparation of –SH/–NH_2_ functionalized MSNs and –NH_2_ functionalized SS-MSNs

MSNs were synthesized using previously published methods.^20^ A description of their synthesis can be found in ESI (Scheme S1†). SS-MSNs were synthesized in a modified way as follows: BTES was added into Solution-1 during the first step of synthesis in the ratio 3.5 (TEOS) : 1 (BTES) (Scheme S1†). As –SH group can interfere with the functionality of BTES, MPTES was not included in the synthesis of SS-MSNs.

### 2.3 Preparation of MSN-SeL, Se-MSNs, and SeNP-MSNs

To create MSN-Se_L_, MSNs (2 mg) were immersed in a 10 mM and 20 mM sodium selenite (Na_2_SeO_3_) water solution (1 mL) for 48 h to prepare MSN-Se_L10_ and MSN-Se_L20_, respectively (Scheme S2†). Se loaded MSNs were collected and washed with ethanol twice and stored in ethanol. To create Se-MSNs, Na_2_SeO_3_ was added during the MSNs synthesis (Scheme S3†). Based on the molar ratios of Se (eqn (1)), 0.177 g, 0.399 g, and 0.684 g Na_2_SeO_3_ powder was added in Solution-2 to synthesize MSNs and SS-MSNs with 10% (Se_10_-MSNs and Se_10_-SS-MSNs), 20% (Se_20_-MSNs) and 30% Se doping (Se_30_-MSNs), respectively. The remaining steps were identical to aforementioned MSNs synthesis procedures.1
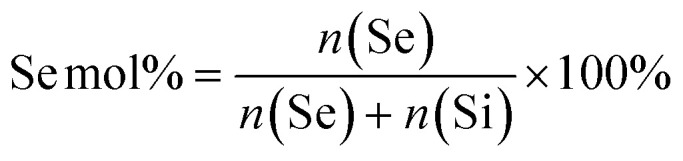


To create SeNP-MSNs, a modified protocol based on a published report was used (Scheme S4†).^28^ In short, CTAB (0.36 g), NH_4_F (0.6 g) and *V*_c_ (1 g) were dissolved in 100 mL milliQ water with constant stirring (1200 rpm) at 80 °C. 2.072 mL (25%; SeNP_25_-MSNs) and 4.144 mL (40%; SeNP_40_-MSNs) Na_2_SeO_3_ water solution (0.25 g mL^−1^) was added dropwise to the solution and left stirring at 1200 rpm and heating at 80 °C for 1 h (eqn (1)). Then, TEOS (1.6794 g) was added dropwise to the mixture and left stirring for another 30 min at RT. The TEOS layer-by-layer assembly, amino functionalization and reflux steps were identical to MSN synthesis procedures.

### 2.4 Characterization of Se-incorporated MSNs

Dynamic light scattering (DLS; Malvern Zetasizer Nano, Panalytical, UK) was used to determine the hydrodynamic sizes and zeta potentials (*ζ*) of synthesized Se-incorporated MSNs. The diameters were measured in ethanol and *ζ* was measured in milliq water (pH 7.0) at 25 °C. The validation of –SH group functionalization was confirmed by labeling MSNs with ATTO-488-maleimide. The fluorescent signal was detected by microplate reader (BIO-RAD microplate reader 550) after mixing ATTO-488 with MSNs overnight. Transmission electron microscopy (TEM; FEI electron microscope, US) was used to image the morphology of Se-incorporated MSNs. All groups of Se-incorporated NPs were homogeneously resuspended in absolute ethanol (1 mg mL^−1^) with sonication, followed by adding on a grid model for TEM imaging after drying out overnight. Attenuated total reflection Fourier transform-infrared spectroscopy (FTIR; Nicolet iS50 spectroscope) was performed to analyze the functional groups of Se-incorporated MSNs. Se-incorporated MSNs resuspended in absolute ethanol were dried out and measured by FTIR. Collected spectra were assessed *via* SpectraGrayph software (version 1.2). Surface area and pore size distribution/volumes of Se-incorporated MSNs were measured by N_2_ adsorption/desorption methods on a Micromeretics ASAP-2060 and calculated by Brunauer–Emmett–Teller (BET) and Barrett–Joyner–Halenda (BJH) methods, respectively.

The total Se incorporation amount in Se-incorporated MSNs was investigated by measuring the Se element level in dissolved samples *via* inductively coupled plasma mass spectrometry (ICP-MS; iCaP Q, Thermo Scientific, US). First, all groups of Se-incorporated MSNs were completely dissolved in aqua regia (0.25 mg NPs dissolved in 1 mL aqua regia), and diluted 20 times in aqueous 1% HNO_3_. 20 ppb Sc was used as internal standard and measured by ICP-MS in standard mode (STD). The measured ions were Se and Si.

### 2.5 Degradation studies

Se release profiles of MSN-Se_L10_, Se_30_-MSNs, and SeNP_40_-MSNs (0.25 mg) in cacodylate buffer ((CH_3_)_2_AsO_2_Na·3H_2_O; Sigma Aldrich GmbH, Germany) at pH 5.0 and 7.4 under stirring (1000 rpm) at 37 °C after incubation for 4, 8, 12, 24, and 72 h, were determined. The supernatants were collected by centrifugation and diluted 20 times in aqueous 1% HNO_3_ with 20 ppb Sc as internal standard. The diluted solution was then measured for Se and Si ion content by ICP-MS in standard mode (STD).

Se release from MSN-Se_L10_, Se_30_-MSNs, and SeNP_40_-MSNs in the presence of glutathione (GSH) and reduced nicotinamide adenine dinucleotide phosphate (NADPH) was investigated using cacodylate buffer that contained GSH (pH 7.4 with 10 mM GSH without NADPH) and that contained GSH and NADPH (pH 7.4 with 10 mM GSH and 1.0 mM NADPH). The supernatants samples were prepared as aforementioned and measured by ICP-MS after dilution.

To assess Se release in cell culture conditions, MSN-Se_L10_, Se_30_-MSNs, and SeNP_40_-MSNs were immersed in cell culture medium (DMEM + 10% FBS) at 0.25 mg mL^−1^ and left for 14 days at 37 °C in static conditions. Afterwards the supernatant was collected, diluted and measured by ICP-MS.

### 2.6 *In vitro* cell cultivation

Saos-2 is a cell line isolated from female osteosarcoma patient with epithelial morphology. Cells were cultured in DMEM (high glucose, l-glutamine, Gibco, US) supplemented with 10% fetal bovine serum (FBS; Gibco, US), 100 U mL^−1^ penicillin and streptomycin (P/S; Gibco, US) at 37 °C, 5% CO_2_ (normal O_2_ level). hFOB 1.19 cell line (ATCC, US) was selected as normal osteoblast cell type as control, which was cultured in DMEM/F12 (Gibco, US) with addition of 10% FBS, 100 U mL^−1^ P/S at 37 °C, 5% CO_2_ (normal O_2_ level).

### 2.7 Cell viability

First, all Se-incorporated MSNs were sterilized by immersion in 70% ethanol for at least 4 h and washed with DMEM or DMEM/F12 cell culture medium. Saos-2 and hFOB cells were seeded in 96-well plates at 1 × 10^5^ cells per well (100 μl) and left for 24 h incubation. After medium exchange, Saos-2 cells were exposed to 1, 5, 25, 50, 75, 100, 150, and 250 μg mL^−1^ NPs concentrations and hFOB cells to 25, 50, 75, and 100 μg mL^−1^ for 24 or 72 h. After exposure, 20 μl of MTS assay (MTS/PMS solution) (Cell titer 96® AQ_ueous_ non-radioactive cell proliferation assay, Promega, US) was added into the wells containing 100 μl of cell culture medium and cells were incubated for another 3 h in the dark. Then the absorbance (O.D.) at *λ*_ex_ = 488 nm and *λ*_em_ = 535 nm was measured using a micro-plate. Cell viability was calculated following eqn (2).2
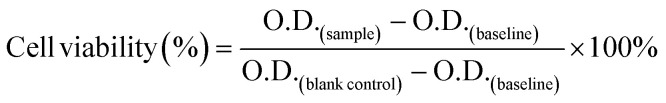
where NPs in regular DMEM medium was marked as baseline, and pure Saos-2 or hFOB cells in regular DMEM medium was marked as blank control. Se free MSNs (MSNs and SS-MSNs) treated Saos-2 cells were marked as negative control.

### 2.8 ROS and apoptosis assays for Saos-2 cells

Saos-2 cells were seeded in 12-well plates at 1 × 10^6^ cells per well (1 mL) and incubated for 24 h. After medium exchange, Saos-2 cells were exposed to 100 μg mL^−1^ MSN-Se_L10_, Se_30_-MSNs, and SeNP_40_-MSNs for 12 h incubation. Cell culture medium was removed and cells were washed by PBS after exposure.

For the ROS assay, 1 mL DCFH/DA (1 μM) was added to each well and left incubating for 30 min in the dark. After that, Saos-2 cells were harvested by trypsinization (0.05% trypsin), counted and resuspended in kit buffer in flow cytometer (FACS) tubes. The fluorescence signal was measured by flow cytometer (AZM BD FACS Canto II, US).

For the apoptosis assay, cells were harvested by 0.05% trypsin in PBS (no EDTA), counted and resuspended in apoptosis buffer. Then 10 μl Annexin V solution and 20 μl PI solution were added in cell suspension for another 15 min incubation at RT in dark. Cells were washed with apoptosis buffer twice and resuspended in the same buffer in FACS tubes. The fluorescence signal was finally visualized by FACS.

### 2.9 Statistical analysis

All results are shown as mean ± SD with at least (*n* = 3). Statistical analysis was performed with GraphPad Prism 9.0 software (US). A 2-way analysis of variance (2-way ANOVA), followed by Tukey's multiple comparison test was used to statistically compare the Se release, cytotoxicity, quantification of ROS fluorescence and apoptosis analyses. Error bars in all figures indicate one standard deviation among the triplicate. * represents *p*-values of significant difference compared to the controls. (*: *p* < 0.05; **: *p* < 0.005; ***: *p* < 0.001; ****: *p* < 0.0001).

## 3. Results

### 3.1 Synthesis and characterization of Se-incorporated MSNs

In this work, three groups of Se incorporated MSNs were developed (Scheme 1). MSN-Se_L_ were created by loading SeO_3_^2−^ in the MSN mesopores and on the surface; Se-MSNs by doping SeO_3_^2−^ in the MSN matrix, and SeNP-MSNs by incorporating SeNP in the core of MSNs.

**Fig. 1:**
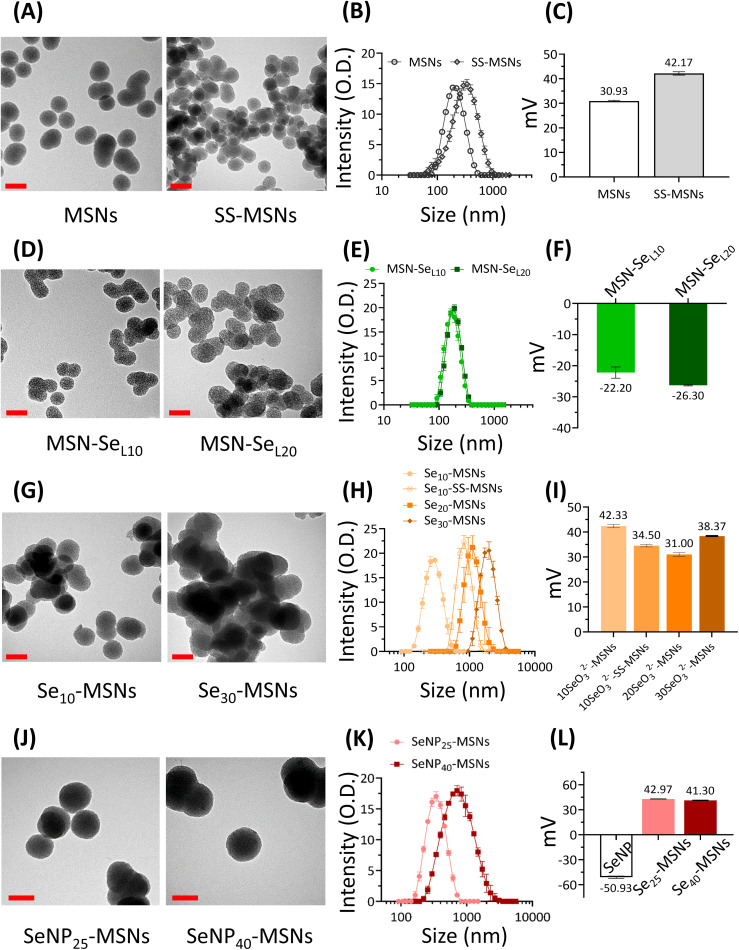
Characterization of Se-incorporated MSNs. (A) TEM images of MSNs and SS-MSNs; scale bar is 100 nm. (B) Hydrodynamic size (measured in ethanol) distribution of MSNs and SS-MSNs. (C) Surface charge of MSNs and SS-MSNs. (D) TEM images of MSN-Se_L10_ and MSN-Se_L20_; scale bars are 100 nm. (E) Hydrodynamic size (measured in ethanol) distribution of MSN-Se_L10_ and MSN-Se_L20_. (F) Surface charge (measured in water) of MSN-Se_L10_ and MSN-Se_L20_. (G) TEM images of Se_10_-MSNs and Se_30_-MSNs; scale bars are 100 nm. (H) Hydrodynamic size (measured in ethanol) distribution of Se_10_-MSNs, Se_10_-SS-MSNs, Se_20_-MSNs, and Se_30_-MSNs. (I) Surface charge (measured in water) of Se_10_-MSNs, Se_10_-SS-MSNs, Se_20_-MSNs, and Se_30_-MSNs. (J) TEM images of SeNP_25_-MSNs and SeNP_40_-MSNs; scale bars are 100 nm. (K) Hydrodynamic size (measured in ethanol) distribution. (L) Surface charge (measured in water) of SeNP_25_-MSNs and SeNP_40_-MSNs. Sample measurements were performed in triplicate.

To develop the NPs, first MSNs functionalized with –NH_2_ groups on the surface and –SH groups in the core (MSN) were synthesized using a previously reported co-condensation method.^20^ MSNs containing a glutathione-cleavable component (BTES) within the silica network (SS-MSNs) were also synthesized. The introduction of BTES creates a redox-sensitive impurity (–S–S–) within the silica network. This impurity is known to induce the redox-dependent degradation of the silica network.^29^ Homogeneous MSNs and SS-MSNs with similar mesoporous structure, spherical shape, and size could be observed *via* TEM and DLS (Fig. 1A and B). To confirm the functionalization of –SH in the core of MSNs, an ATTO-488 dye containing maleimide, which can bind to –SH, was used to fluorescently label MSNs. A high fluorescent intensity could be observed (Fig. S1†). The same reaction was performed with SS-MSNs, but since these did not contain free thiols in the core of the structure, fluorescent labeling of these nanoparticles was unsuccessful (Fig. S1†). High positive zeta surface charge as measured by DLS validated the –NH_2_ surface functionalization of both MSNs and SS-MSNs (Fig. 1C). The results confirmed the successful synthesis of MSNs (–SH_in_–NH_2 out_) and SS-MSNs (–NH_2 out_).

To create MSN-Se_L_, MSNs were immersed in 10 mM and 20 mM Se solutions for 48 h to create MSN-Se_L10_ and MSN-Se_L20_, respectively. The morphology of MSN-Se_L_ showed a less clear mesoporous structure and displayed higher contrast compared to MSNs (Fig. 1D). SeO_3_^2−^ loading did not change the size of the NPs (Fig. 1E; Table S1; Fig. S2†). A shift in surface charge from positive (+30.93 ± 0.29 mV for MSNs) to negative (−22.20 ± 1.81 mV for MSN-Se_L10_ and −26.30 ± 0.2 mV for MSN-Se_L20_) was observed after SeO_3_^2−^ loading, indicating the incorporation of the negatively charged selenite on the surface was successful (Fig. 1F).

Se was doped in the silica matrix by adding SeO_3_^2−^ during MSN synthesis to develop Se-MSNs. Increasing the Se doping ratio from 10 to 30 mol% resulted in significantly larger hydrodynamic sizes and particle aggregation, as observed by DLS measurements (Fig. 1H; Table S1†). Moreover, 20 and 30% doping resulted in altered NP morphology; a less spherical shape with a less clear mesoporous structure was observed (Fig. 1G; Fig. S3†). SS-MSNs containing 10% of Se doping resulted in significantly larger hydrodynamic sizes compared to MSNs doped with 10% Se (Fig. S3; Table S1†). Unchanged surface charge revealed that Se doping did not affect the MSNs surface properties (Fig. 1I).

To synthesize SeNP-MSNs, SeNPs of approximately 30–60 nm in size were synthesized *via* a redox reaction between SeO_3_^2−^ and ascorbic acid (*V*_c_) (Fig. S2 and S4†), followed by surface grafting of a mesoporous silica coat. SeNP-MSNs with a darker inner core and a clear mesoporous structure on the surface could be observed using TEM (Fig. 1J; Fig. S4†). Silica coating on SeNPs resulted in significantly larger NP sizes compared to MSNs and SeNPs (Fig. 1J, K; Fig. S2†). Increasing the SeNPs amount from 25 mol% (SeNP_25_-MSNs) to 40 mol% (SeNP_40_-MSNs) further increased NP size (Fig. 1K; Fig. S2†). A shift in surface charge from negative (−50.93 ± 1.4 mV for SeNPs) to positive (+42.97 ± 0.2 mV for SeNP_25_-MSNs and +41.30 ± 0.4 mV for SeNP_40_-MSNs) was observed after mesoporous silica coating of SeNPs (Fig. 1L). All groups of Se-incorporated MSNs were homogeneous with Polydispersity indices (Pdi) below 0.35 (Table S1†).

The Se-incorporated MSNs were further characterized using FTIR (Fig. S5†). Bands at 447 cm^−1^ (Si–O–Si bending), 794 cm^−1^ and 1053 cm^−1^ (Si–O–Si stretching vibration), and 957 cm^−1^ (Si–OH stretching vibration) (blue area) could be observed in all synthesized NPs resulting from the silica matrix.^30^ Small bands at 1631 cm^−1^ (green area) and 2923 cm^−1^ (yellow area) were assigned to –NH_2_ stretching vibrations and C–H stretching vibrations from the APTES,^31^ proving the amino-functionalization was successful. In the FTIR spectra of MSN-Se_L_ (Fig. S5B†), these bands were not as clearly visible, possibly due to the SeO_3_^2−^ loading.

Mesopore parameters of as-synthesized Se-incorporated MSNs were determined by N_2_ adsorption. To investigate the effect of the three different modes of Se incorporation on surface area and pore size, MSN, MSN-Se_L10_, Se_30_-MSNs, and SeNP_40_-MSNs were analysed. Pore sizes were in the range of 2.90 to 3.27 nm as determined by BJH method (Table S2†). Thus, surface Se loading, matrix Se doping and SeNP incorporation didn't change mesopore diameters. However, SeNP_40_-MSNs had significantly decreased pore volume (0.185 cm^3^ g^−1^) compared to MSNs, MSN-Se_L10_, and Se_30_-MSNs ( Table S3†). Also the specific surface for SeNP_40_-MSNs was significantly lower (228.9 cm^3^ g^−1^) compared to MSNs, MSN-Se_L10_, and Se_30_-MSNs (between 746 and 964 cm^3^ g^−1^, Table S4†).

**Fig. 2:**
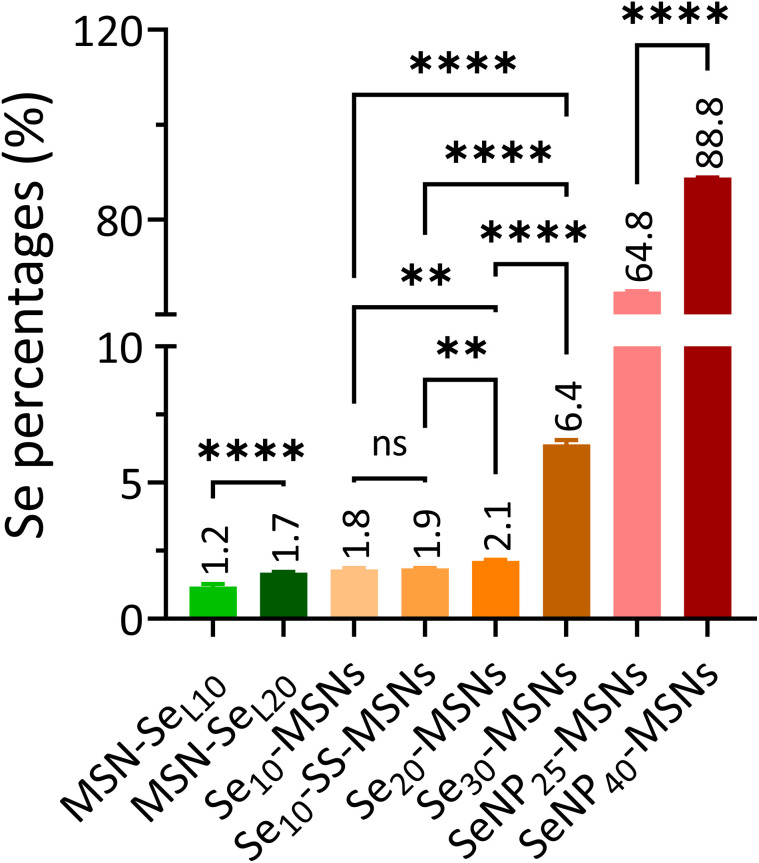
Se content (Se mol%) in MSN-Se_L_, Se-MSNs and SeNP-MSNs using ICP-MS analysis. *n* = 3. * represents *p*-values of significant difference compared to the controls. (*: *p* < 0.05; **: *p* < 0.005; ***: *p* < 0.001; ****: *p* < 0.0001).

In order to investigate the Se incorporation efficiency, ICP-MS was used to detect the total amount of Se and Si present (Fig. 2). For MSN-Se_L_, increasing the SeO_3_^2−^ concentration (from 10 to 20 mM) led to an obvious increase of Se incorporation (Se mol%) from 1.19% for MSN-Se_L10_ to 1.70% for MSN-Se_L20_. For Se-MSNs, doping higher molar rates of Se, also resulted in higher Se incorporation; 1.81% for Se_10_-MSNs, 2.13% for Se_20_-MSNs, and 6.41% for Se_30_-MSNs. Adding a BTES linker in Se_10_-MSNs (Se_10_-SS-MSNs) did not affect the Se doping percentages (Se: 1.85%). SeNP-MSNs contained the highest Se amount. Specifically, SeNP_25_-MSNs contained 64.84% of Se and SeNP_40_-MSNs 88.84% of Se. In summary, three groups of Se-incorporated MSNs were successfully synthesized, with various amounts of Se incorporation amounts.

### 3.2 Ion release profile and stability of Se-incorporated MSNs

**Fig. 3:**
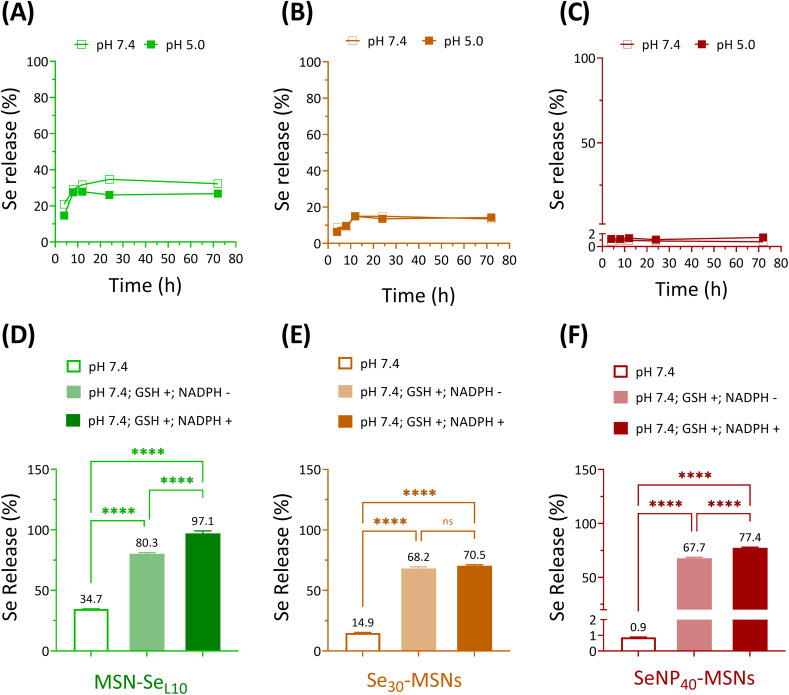
Se release of (A) MSN-Se_L10_, (B) Se_30_-MSNs and (C) SeNP_40_-MSNs in pH 7.4 and pH 5.0 at 4, 8, 12, 24, and 72 h incubation. Se release profiles of (D) MSN-Se_L10_, (E) Se_30_-MSNs and (F) SeNP_40_-MSNs after 24 h incubation in the absence/presence of GSH (10 mM) and NADPH (1.0 mM) at neutral conditions. *n* = 3. * represents *p*-values of significant differences compared to the controls. (*: *p* < 0.05; **: *p* < 0.005; ***: *p* < 0.001; ****: *p* < 0.0001).

Next, Se release rates were investigated in acidic (5.0) and neutral (7.4) pH conditions using cacodylate buffer. Ca and P-free cacodylate buffer was used to avoid calcium phosphate deposition on MSNs, which might interfere the degradation of Se-incorporated MSNs.^20^ MSN-Se_L10_, Se_30_-MSNs, and SeNP_40_-MSNs were selected for these studies. Fast Se release was observed for MSN-Se_L10_ and Se_30_-MSNs, where maximum Se release was observed after 12 h incubation in both pH conditions (Fig. 3A and B). No Se release was observed for SeNP-MSNs (Fig. 3C). Interestingly, no difference in release rate as a function of pH was observed for any of the three NPs (Fig. 3A–C). The amount of Se released was 752.7 ppb for MSN-Se_L10_, 546.5 ppb for Se_30_-MSNs, and 606.7 ppb for SeNP_40_-MSNs after 72 h (Fig. S6A–C†). This amounted to 35%, 15% and less than 2% of total Se amount in MSN-Se_L10_, Se_30_-MSNs and SeNP_40_-MSNs, respectively (Fig. 3A–C). Moreover, Se release in cell culture medium after 14 days incubation was 10.27%, 9.92% and 0.3% for MSN-Se_L10_, Se_30_-MSNs, and SeNP_40_-MSNs, respectively (Fig. S7†).

Se is known to be reduced by GSH/NADPH.^32^ Therefore, we investigated whether the presence of GSH/NADPH can induce Se release from the synthesized MSNs. MSN-Se_L10_, Se_30_-MSNs, and SeNP_40_-MSNs were incubated with known intracellular concentrations of GSH (10 mM) and NADPH (1 mM),^33^ and Se release profile was investigated after 24 h using ICP-MS. Se-incorporated MSNs in neutral buffer (without the presence of GSH and NADPH) were included as negative controls. Se release significantly increased in the presence of GSH and NADPH for all three NPs tested (Fig. 3D–F). Specifically, Se release increased from 34.7% to 80.3% in the presence of GSH (*p* < 0.0001) (1741.6 ppb) and to 97.1% in the presence of both GSH and NADPH (*p* < 0.0001; 2107.5 ppb) for MSN-Se_L10_ (Fig. S8A†). For Se_30_-MSNs, Se release increased from 14.9% to 68.2% in presence of GSH (*p* < 0.0001; 2494.9 ppb) and 70.5% when both GSH and NADPH were present (*p* < 0.0001; 2578.5 ppb) (Fig. S8B†). For SeNP_40_-MSNs, Se release remarkably increased from 0.9% to 67.7% (GSH only; *p* < 0.0001; 47 283.7 ppb) and 77.4% (both GSH and NADPH; *p* < 0.0001; 54 069.4 ppb) (Fig. S8C†). MSNs remained stable in buffer conditions after 24 h incubation period, also in the presence of GSH/NADPH (Fig. S9A and D†). In contrast, TEM images of MSN-Se_L10_ after exposure to GSH/NADPH revealed a clear mesoporous structure, indicating Se loaded on the surface and mesopores was released (Fig. S9B and E†). Moreover, TEM images of Se_30_-MSNs and SeNP_40_-MSNs showed a clear sign of degradation after 24 h incubation in buffer containing GSH/NADPH (Fig. S9C, D, F and G†).

In summary, Se release in the presence of GSH and NADPH was observed for all tested Se-incorporated MSNs.

### 3.3 Cytotoxicity of Se-incorporated MSNs

**Fig. 4:**
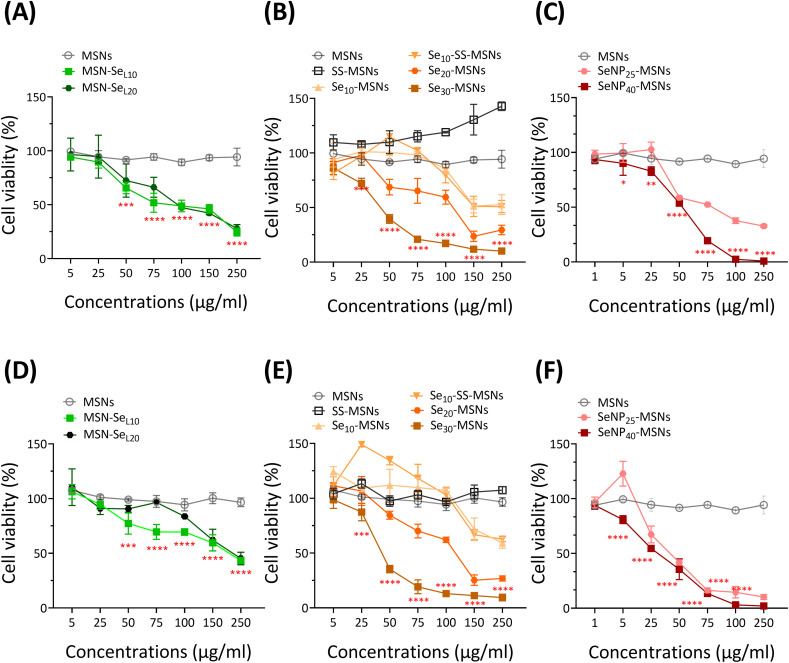
Viability of Saos-2 cells after 24 h-exposure to (A) MSN-Se_L10_, (B) Se_30_-MSNs, (C) SeNP_40_-MSNs, and after 72 h-exposure to (D) MSN-Se_L10_, (E) Se_30_-MSNs and (F) SeNP_40_-MSNs. *n* = 3. * represents *p*-values of significant difference compared to the controls. (*: *p* < 0.05; **: *p* < 0.005; ***: *p* < 0.001; ****: *p* < 0.0001).

Next, the cytotoxic effect of the synthesized Se-incorporated MSNs on Saos-2 cells was investigated using the MTS assay. MTS assay measures cell metabolism, and a decrease of absorbance indicates NPs cytotoxicity. Saos-2 cell line was established from an 11-year-old Caucasian female with osteogenic sarcoma in 1973 (ATCC), which has been widely used as a OS cell candidate in published research.^34^ Saos-2 cells treated with MSNs and SS-MSNs (negative control groups) showed no significant toxicity even at high concentrations (250 μg mL^−1^), illustrating Se-free MSNs are not cytotoxic in the concentration ranges we tested. All synthesized Se-incorporated MSNs showed dose-dependent effects on Saos-2 cell viability after 24 and 72 h of incubation (Fig. 4). The concentrations of MSN-Se_L10_, Se_30_-MSNs, and SeNP_40_-MSNs needed to induce 25% (IC_25_), 50% (IC_50_) and 75% (IC_75_) cell viability loss are shown in Table 1. After 24 h-exposure, SeNP_40_-MSNs and Se_30_-MSNs were most cytotoxic with IC_50_ values between 40–50 μg mL^−1^ compared to 71 μg mL^−1^ for MSN-Se_L10_. After 72 h, SeNP_40_-MSNs and Se_30_-MSNs exhibited lower IC_50_ values compared to those after 24 h, with SeNP_40_-MSNs being most effective (Table 1). There was no significant difference in toxicity between MSN-Se_L10_ and MSN-Se_L20_, probably due to their similar Se loading amounts (Fig. 4A and D). In contrast, higher doping ratios in Se-MSNs resulted in higher cytotoxicity after 24 h-exposure, but not after 72 h-exposure.

**Table 1:** IC_25_, IC_50_, and IC_75_ values of Saos-2 cells exposed to MSN-Se_L10_, Se_30_-MSNs or SeNP_40_-MSNs for 24 and 72 h

Times	Samples	IC_25_ (μg mL^−1^)	IC_50_ (μg mL^−1^)	IC_75_ (μg mL^−1^)
24 h	MSN-SeL_10_	34	71	148
	Se_30_-MSNs	29	42	62
	SeNP_40_-MSNs	42	55	72
72 h	MSN-SeL_10_	84	—	—
	Se_30_-MSNs	30	40	53
	SeNP_40_-MSNs	18	36	69

Low concentrations of SS-MSNs and Se_10_-SS-MSNs led to increased activity. Both SS-MSNs, Se_10_-SS-MSNs contain a degradable matrix due to incorporation of a redox responsive –S–S– bond, and Si ion release can lead to increased metabolic activity.^35^ Furthermore, no significant differences in toxicity were observed between Se_10_-MSNs and Se_10_-SS-MSNs (Fig. 4B and E), demonstrating that (faster) silica matrix degradation did not lead to higher cytotoxic effect of the NPs. SeNP_40_-MSNs were more cytotoxic compared to SeNP_25_-MSNs after 24 h-exposure but not after 72 h-exposure (Fig. 4C and F). In summary, all synthesized Se-MSNs were able to induce Saos-2 cell death in a concentration dependent manner, where Se_30_-MSNs, and SeNP_40_-MSNs showed the highest potency.

**Fig. 5:**
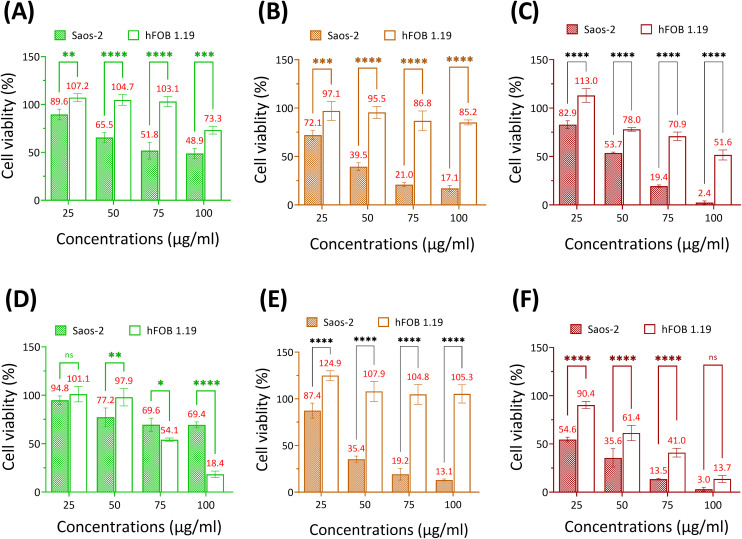
Viability of Saos-2 and hFOB cells after 24 h-exposure to (A) MSN-Se_L10_, (B) Se_30_-MSNs, (C) SeNP_40_-MSNs, and after 72 h-exposure to (D) MSN-Se_L10_, (E) Se_30_-MSNs and (F) SeNP_40_-MSNs. *n* = 3. * represents *p*-values of significant difference compared to the controls. (*: *p* < 0.05; **: *p* < 0.005; ***: *p* < 0.001; ****: *p* < 0.0001).

Se-incorporated MSNs with the highest cytotoxicity from each group, *i.e.*, MSN-Se_L10_, Se_30_-MSNs, and SeNP_40_-MSNs, were selected and their cytotoxicity towards osteoblast (hFOB 1.19) investigated. The three tested NPs were significantly more cytotoxic for Saos-2 cells than for hFOB cells (Fig. 5). Specifically, hFOB cells exposed to 50 or 75 μg mL^−1^ MSN-Se_L10_ for 24 h showed no loss of viability, whereas a decrease in Saos-2 viability of respectively 34.5% and 49.2% was observed (Fig. 5A). However, after 72 h-exposure, higher toxicity of MSN-Se_L10_ towards hFOB cells compared to Saos-2 cells was observed (Fig. 5D). Se_30_-MSNs showed high selectivity; 24 h-exposure to 50–100 μg mL^−1^ of NPs did not have a significant cytotoxic effect on hFOB cells, whereas, 60.5% (50 μg mL^−1^) and 82.9% (100 μg mL^−1^) cell viability loss was observed in Saos-2 cells (Fig. 5B). This selectivity was still present after 72 h of NP exposure (Fig. 5E). Exposure to SeNP_40_-MSNs also showed differential activity; 22.0% (50 μg mL^−1^), 29.1% (75 μg mL^−1^), and 48.4% (100 μg mL^−1^) cell viability loss was observed in hFOB cells. While exposure to the same concentrations led to a higher cell viability loss in Saos-2 cells (50 μg mL^−1^ led to 46.3%, 75 μg mL^−1^ led to 80.6% cell loss, and 100 μg mL^−1^ led to 97.4% cell loss;Fig. 5C). However, this differential selectivity was partly lost after 72 h-exposure (Fig. 5F). In summary, MSN-Se_L10_, Se_30_-MSNs, and SeNP_40_-MSNs demonstrated a significantly higher cytotoxic effect towards Saos-2 cells compared to hFOB 1.19 cells, where Se_30_-MSNs displayed the highest selectivity.

### 3.4 ROS accumulation and apoptotic effect caused by Se incorporated MSNs

**Fig. 6:**
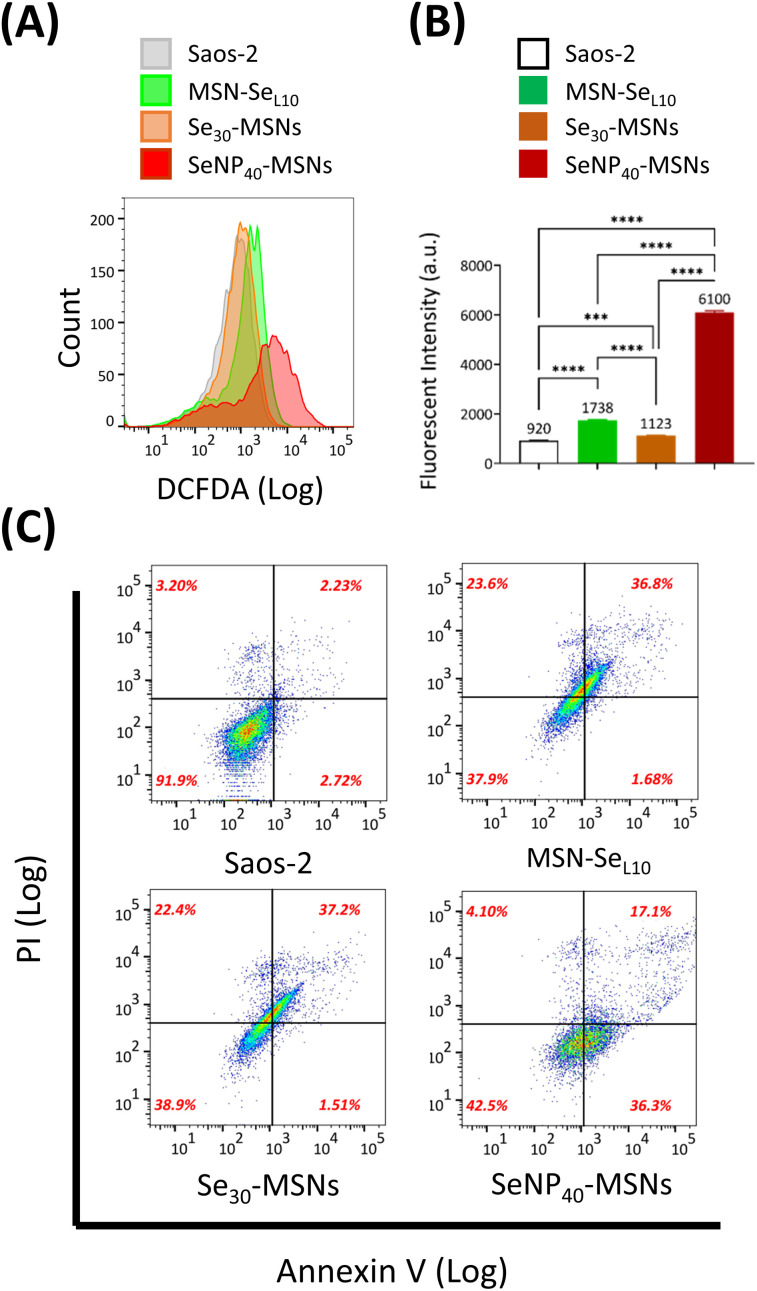
ROS and apoptosis assay. (A) Histograms and (B) fluorescent intensity of Saos-2 cells exposed to 100 μg mL^−1^ MSN-Se_L10_, Se_30_-MSNs and SeNP_40_-MSNs after 12 h incubation *n* = 3. * represents *p*-values of significant difference compared to the controls. (*: *p* < 0.05; **: *p* < 0.005; ***: *p* < 0.001; ****: *p* < 0.0001). (C) Double-labelled (annexin V/PI) FACS dot plots of Saos-2 cells exposed to MSN-Se_L10_, Se_30_-MSNs and SeNP_40_-MSNs (100 μg mL^−1^) after 12 h incubation.

Se is known to induce ROS and subsequent cell apoptosis.^36^ Hence, we surmised that the low cell viabilities of Saos-2 cells and selective cytotoxicity were attributed to Se-induced ROS generation and subsequent cell apoptosis. The ability of MSN-Se_L10_, Se_30_-MSNs, and SeNP_40_-MSNs to induce ROS in Saos-2 cells was evaluated using the DCFDA assay. DCFDA can be oxidized to a fluorescent product 2,7-dichlorofluorescein (DCF) by generated intracellular ROS. Fluorescence of DCF was analyzed using flow cytometry (Fig. 6A and B). A peak shift as a result from DCF fluorescence was observed after treatment with all three NPs but it was most pronounced for cells exposed to SeNP_40_-MSNs (Fig. 6A). ROS levels of Saos-2 cells after 12 h exposure to Se-incorporated MSNs followed the trend Se_30_-MSNs < MSN-Se_L10_ < SeNP_40_-MSNs (Fig. 6B). Only slight shifts in the DCF histograms were observed in cells exposed to MSN-Se_L10_ and Se_30_-MSNs (Fig. 6B). To assess whether the selected Se-incorporated MSNs could induce apoptosis, Annexin V/PI assay was used (Fig. 6C). All tested Se-incorporated MSNs (MSN-Se_L10_, Se_30_-MSNs, and SeNP_40_-MSNs; 100 μg mL^−1^) induced apoptosis after 12 h incubation (Fig. 6C; Fig. S10†). Specifically, the population of MSN treated Saos-2 cells moved to the upper-right quadrant compared to control group, indicating that a significant proportion of cells were in late apoptosis stage. In summary, after 12 h-exposure, all tested Se-incorporated MSNs could significantly induce ROS generation and in turn, trigger cell apoptosis. SeNP_40_-MSNs showed the highest effect at this time point.

## 4. Discussion

Chemotherapeutics based on inorganic Se have demonstrated promising anti-cancer properties.^37^ The effectiveness of inorganic Se is, however, highly dependent on dose and its oxidation state.^38^ Moreover, the cellular uptake of inorganic Se is generally slow,^39^ which can result in extracellular Se accumulation hindering the selective therapeutic efficiency of OS. Thus, for its effective application in cancer therapy, (selective) intracellular Se delivery is needed. MSNs are promising vehicles for Se delivery because they are considered biocompatible and can provide a protective matrix for Se delivery, to transport Se into cells in a dose-controlled manner.^19^ Moreover, MSNs have inherent bone regenerative capabilities,^19^ which make them especially interesting for application in OS. Due to inorganic crystal framework and mesoporous structure of MSNs, Se can be doped within the matrix, loaded into the mesopores or incorporated in the core structure as NPs. In this work, we reported the successful synthesis of Se-incorporated MSNs *via* these three distinct methods, and investigated the effect of Se incorporation mode on incorporation efficiency, release and cancer cell cytotoxicity.

Se loading onto the MSN surface and into the mesopores was successfully achieved. Incubating MSNs in high SeO_3_^2−^ solution (*i.e.* 10 mM) did not lead to higher Se loading in MSNs. This indicated that Se loading had reached a plateau. Compared to the other two groups, SeO_3_^2−^ loading resulted in lowest Se incorporation rates. Similar incorporation efficiency was observed in a study loading Sr within the mesopores of MSNs.^20^

Se doping in the silica network was successfully achieved by including Se salts during the co-condensation reaction. To the best of our knowledge, we are the first to report successful SeO_3_^2−^ doping into MSN matrix. There are several reports on doping SeO_3_^2−^ into calcium phosphate and bioglass NPs and doping of other (similar) ions, such as Cu^2+^, Sr^2+^, and Fe^3+^ into MSNs.^22,24,25,40–42^ We were also able to dope Se into the MSN matrix that contained a BTES degradable unit, showing that ion doping is compatible with organosilanes silica matrix doping. Furthermore, we showed that we could increase the doping ratio by increasing the amount of Se precursor (Na_2_SeO_3_) during NP synthesis from 2 to approximate 7 mol% Se doping. The maximal doping efficiency was approximately 20%, whereas higher Se doping affected MSN structure including their shape and homogeneity. Similar ion doping efficiency into the MSN matrix was observed in a previous study using strontium (Sr).^25^

We successfully coated SeNP with MSNs in a core/shell structure *via* a modified *in situ* synthesis method.^28^ The amount of Se that we were able to incorporate using this method was significantly higher (10–74 fold higher) compared to the other modes of Se incorporation. Several studies reported on the development of core/shell MSNs based on gold (Au) or Fe_3_O_4_ NPs as cores, for cancer diagnosis and therapy.^43^ However, no other studies have looked into SeNP MSN core/shell NPs for inhibiting OS cells, as we have presented here.

Se-incorporated MSNs degraded in the presence of GSH/NADPH and only to a limited extent in neutral or acidic conditions in the absence of GSH/NADPH. No significant difference in Se release was observed as a function of pH. This is in contrast to previously reported studies using Se-incorporated calcium phosphate NPs, where rapid Se release was shown in acidic conditions (pH 5.0).^44^ This could be due to the higher stability of the silica network in acidic conditions compared to calcium phosphate.^28^ It has been reported that Se^4+^ (SeO_3_^2−^) and Se^0^ (SeNP) can be reduced *via* redox reactions in the presence of GSH and NADPH,^32^ which can explain the sensitivity of our Se-incorporated MSNs in these conditions leading to Se release. The GSH/NADPH concentrations we used are similar to reported intracellular levels, indicating that MSNs mediate rapid Se release upon entering into cells, while remaining stable at neutral conditions.

All three groups of Se-incorporated MSNs were cytotoxic towards Saos-2 cells in a dose-dependent manner. The toxicity is likely caused by redox reactions between Se (both Se^4+^ and Se^0^) and GSH/NADPH, where ·O_2_^−^ is produced, increasing intracellular ROS.^32^ ROS accumulation induced by Se^4+^ (SeO_3_^2−^) or Se^0^ (SeNP) is known to enable the activation of ROS-mediated cell death pathways (*e.g.*, apoptosis, autophagy, and ferroptosis).^22,32,45^

MSN-Se_L10_, Se_30_-MSNs, and SeNP_40_-MSNs were more cytotoxic towards Saos-2 cells than towards hFOB cells. Of note, ROS levels are generally higher in OS cells compared to normal bone cells.^46^ Further increases of ROS may thus exceed the threshold in OS cells, while in osteoblasts, ROS remains at safe levels.^15^ Se doped MSNs showed the highest selectivity; they significantly reduced Saos-2 viability, while FOB cells remained unaffected. Based on Se release in buffer containing GSH/NADPH, Se doping in MSN matrix displayed a lower percentage of Se release (70.5%) compared to MSN-Se_L10_ (97.1%) and SeNP_40_-MSNs (77.4%), indicating incomplete Se release after 24 hours for Se_30_-MSNs and SeNP_40_-MSNs. Moreover, the Se content in these particles was higher than in Se loaded MSNs but lower than in SeNP-MSNs, suggesting that there may be an optimal concentration range for Se to allow selective OS therapy. High selective toxicity of Saos-2 cells was also observed after exposure to SeNP-MSNs after 24 h, however, after 72 h, hFOB cells were also highly effected. This is likely related to the high Se incorporation (25% and 40 mol%) in these particles compared to the Se doped and loaded MSNs. Considering that it was reported that SeNP can efficiently generate ROS,^32^ longer exposure times may lead to more toxicity also in hFOB cells. MSN-Se_L_ showed the lowest cytotoxic effect towards Saos-2 cells, which is probably due to a relatively low amount of incorporated Se. Moreover, it is possible that lower amounts of MSN-Se_L_ entered Saos-2 *via* endocytosis compared to Se-MSNs and SeNP-MSNs, because of its lower surface charge, making it less favorable for cellular uptake.^47^

The ROS level assays validated that MSN-Se_L10_, Se_30_-MSNs, and SeNP_40_-MSNs led to elevation of ROS in Saos-2 cells after 12 h-exposure. This is in line with other studies that also showed that released Se could induce ROS.^21,40,41^ SeNP_40_-MSNs showed the greatest effect on producing ROS at this time point, which could be explained by the fact that there was a higher amount of Se in SeNP-MSNs than in MSN-Se_L10_ and Se_30_-MSNs, and that SeNP (Se^0^) can generate ROS with higher efficiency than SeO_3_^2−^ (Se^4+^).^32^ We also showed that MSN-Se_L10_, Se_30_-MSNs, and SeNP_40_-MSNs could induce apoptosis of Saos-2 cells after 12 h-exposure. It is likely that the cell apoptosis was triggered by generated ROS. Indeed it has been reported that ROS induced by Se^4+^ (SeO_3_^2−^) can initiate apoptosis.^36,44^ Taken together, our ROS and apoptosis assay indicate that Se-incorporated MSNs inhibit Saos-2 cells *via* a ROS mediated apoptosis pathway.

The herein developed Se incorporated MSNs are promising for both systemic targeting and localized OS therapy due to the selective anti-OS property of Se and the tunable surface properties of MSNs. For example, targeting ligands can be easily conjugated to enhance the selectivity/targeting of the MSNs. Moreover, additional drugs or growth factor can be introduced in the mesopores for combination therapy. The nanosize of the Se-incorporated MSNs also allows facile embedding in bone regenerative microparticles/macroscaffolds, which can be used as bifunctional composites that can treat OS locally and simultaneously regenerate bone.

## 5. Conclusions

In this work, we developed MSNs as new delivery carriers for chemotherapeutic Se compounds. To analyse how MSNs can be best utilized for Se delivery, Se was incorporated *via* three methods, with varied incorporation amount, Se oxidation state and release rate. Specifically, three series of MSNs were successfully developed by (1) loading SeO_3_^2−^ on mesopores/surface of MSNs (MSN-Se_L_), (2) doping SeO_3_^2−^ into MSN matrix (Se-MSNs), and (3) incorporating SeNP into MSN framework (SeNP-MSNs). We are the first to report successful doping of Se in the silica matrix of MSNs. Moreover, we were able to increase the Se doping ratio to up to 7 mol% without negatively affecting nanoparticle formation. Se loading in the mesopores and on the surface led to the lowest incorporation amounts, while high Se incorporation of up to 88% was observed in SeNP-MSNs. All synthesized Se-incorporated MSNs were stable in neutral conditions but rapidly degraded in the presence of GSH/NADPH, indicating that our Se-incorporated MSNs are controllably degraded once internalized inside cells, while remaining stable in extracellular surroundings where GSH/NADPH levels are relatively low. All synthesized Se-incorporated MSNs displayed dose-dependent inhibition of Saos-2 cell viability, with Se_30_-MSNs and SeNP_40_-MSNs having the strongest effect. More importantly, all developed MSNs showed a higher selectivity towards cancer cells compared to healthy osteoblasts, with Se_30_-MSNs showing the highest selectivity. Moreover, exposure to the three types of Se-MSN led to the production of ROS and induction of apoptosis.

## Conflicts of interest

There are no conflicts to declare.

## Supplementary Material

BM-011-D2BM02102A-s001
