# Supplementary material for: Selenium-incorporated mesoporous silica nanoparticles for osteosarcoma therapy
Source: Biomater Sci. 2023 Apr 19;11(11):3828–39. doi: 10.1039/d2bm02102a (PMC10227887; doi:10.1039/d2bm02102a)
Supplement: BM-011-D2BM02102A-s001 [file BM-011-D2BM02102A-s001.pdf]

Electronic Supplementary Material (ESI) for Biomaterials Science.
This journal is © The Royal Society of Chemistry 2023

Selenium-incorporated Mesoporous Silica Nanoparticles for Osteosarcoma Therapy

Lei He, Pamela Habibovic and Sabine van Rijt*

Department of Instructive Biomaterials Engineering, MERLN Institute for Technology Inspired Regenerative Medicine, Maastricht University, P.O. Box 616, 6200 MD Maastricht, The Netherlands.

**E-mail: s.vanrijt@maastrichtuniversity.nl*

1. Methods

1.1 Preparation of –SH/–NH_2 functionalized MSNs

Briefly, two solutions containing tetraethyl orthosilicate (TEOS; 1.63 g), 3-mercaptopropyl triethylsilane (MPTES; 112 mg), triethanolamine (TEA; 14.3 g) (Solution-1), and cetyltrimethylammonium chloride (CTAC 25 wt% in H_2O; 2.41 ml), ammonium fluoride (NH_4F; 100 mg), H_2O (millQ water; 21.7 g) (Solution-2) were heated under static condition (for Solution-1) and constant stirring speed (500 rpm) (for Solution-2) in oil bath at 90 °C and 60 °C respectively for 20 min. Solution-2 was then rapidly poured into Solution-1 with constant stirring at 1000 rpm for 20 min without heating. TEOS (138.3 mg) was added to this mixture in four times every 3 min (≈ 37 µl every time) and the mixture was left stirring for 30 min at room temperature (RT). Subsequently, a mixture of TEOS (19.3 mg) and 3-aminopropyl triethoxysilane (APTES; 20.5 mg) were added into the emulsion and left stirring overnight at RT with constant stirring (1000 rpm). Then, the NPs were collected and washed with absolute ethanol twice by centrifugation. To remove the template, NPs suspension were washed through a 2-step reflux. First, NPs were resuspended in ammonium nitrate ethanoic solution (NH_4NO_3; 2 g in 100 ml ethanol) heated at 90 °C for 45 min. Second, NPs washed by ethanol were resuspended in hydrochloric acid (37% HCl) ethanoic solution (10 ml HCl and 90 ml ethanol) heated at 90 °C for another 45 min. Finally, refluxed MSNs were collected and washed with ethanol triple times and stored for further use.

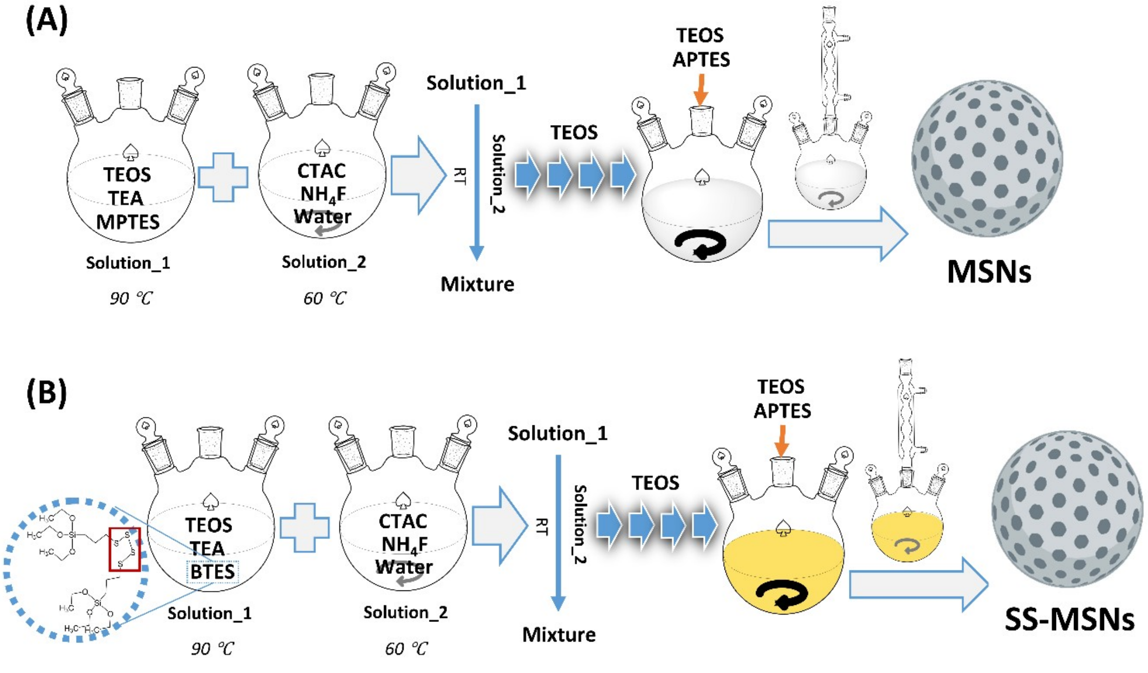

Scheme S1 (A) MSNs and (B) SS-MSNs synthesis procedures.

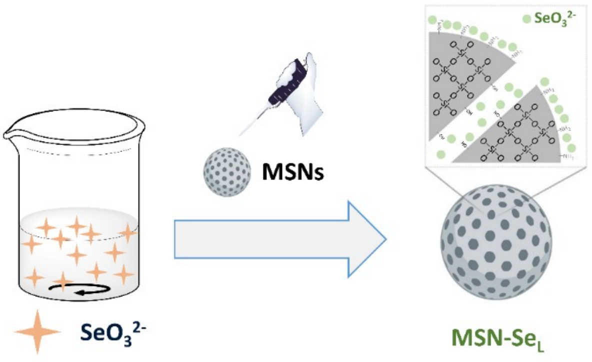

Scheme S2 MSN with Se loading (MSN-Se$_L$) synthesis procedures.

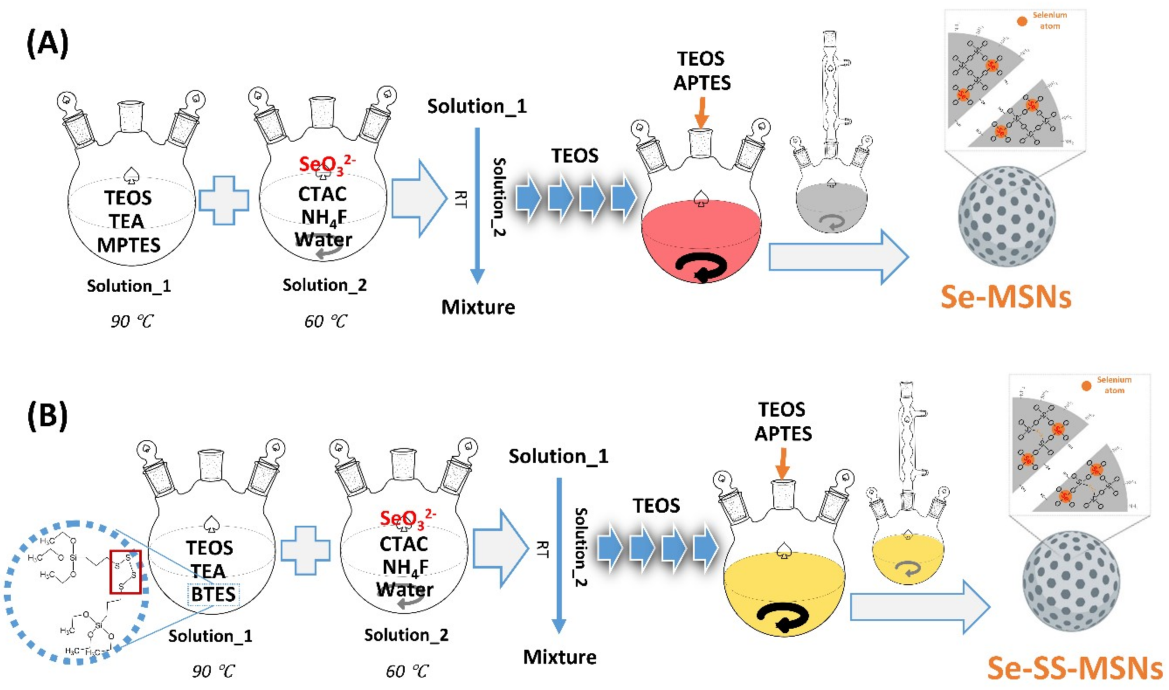

Scheme S3 (A) Se-doped MSNs (Se-MSNs) and (B) Se-doped MSNs incorporated with –S–S– linkers (Se-SS-MSNs) synthesis procedures.

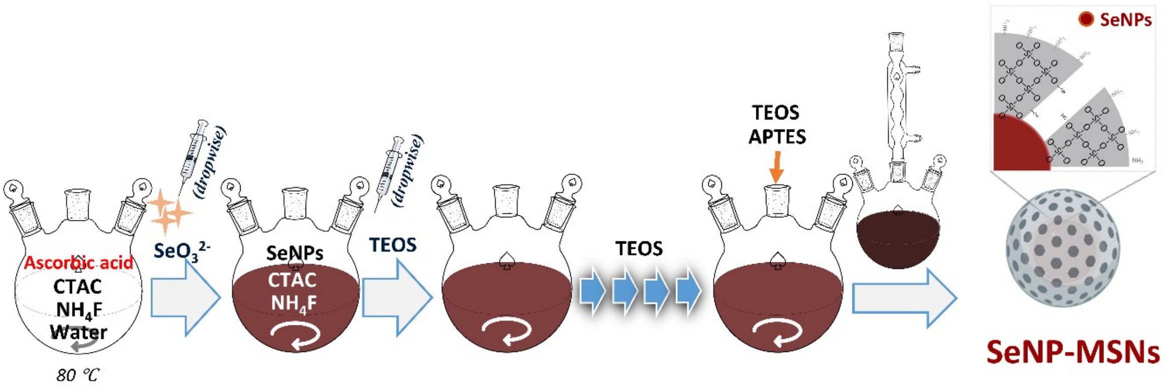

Scheme S4 SeNP incorporated MSNs (SeNP-MSNs) synthesis procedures.

1.2 Doping efficiency of Se-doped MSNs

Doping efficiency refers to the efficiency of Se doping by comparing exact Se doping ratios with theoretical Se doping ratios. As we tested the real incorporation amount of Se in Se-

doped MSNs, we can calculate the doping efficiency by comparing the practical Se doping amount to theoretical Se doping amount. The calculation equation was listed as below.

$$Se\ doping\ efficency\ \% = \frac{Practical\ Se\ doping\ rates\ (mol\%)}{Theoretical\ Se\ doping\ rates\ (mol\%)} \times 100\%$$ **(Equation S1)**

2. Results

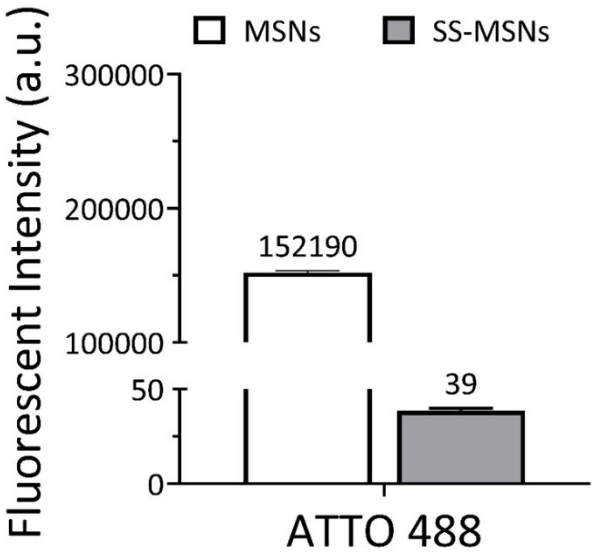

Fig. S1 Atto-488 fluorophore labeling manifested the existence of –SH groups in MSNs but not in SS-MSNs.

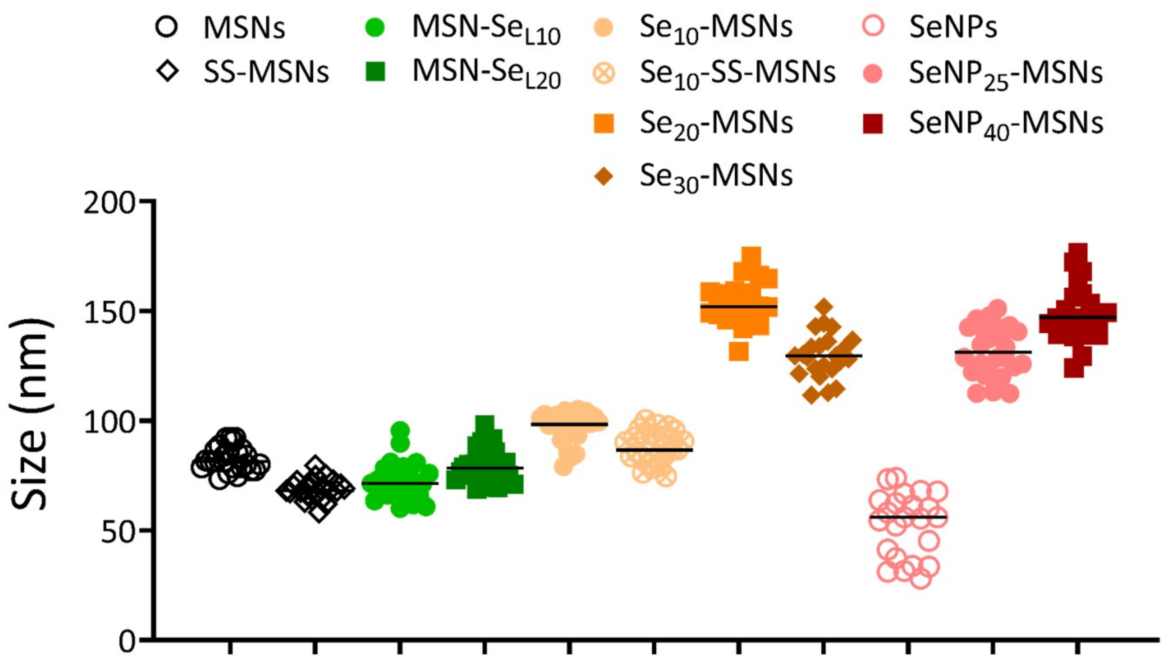

Fig. S2 Size distribution of all groups of Se-incorporated MSNs calculated by image J software (US). The calculated object number is 25 (n = 25). The blank lines represent the median of each group.

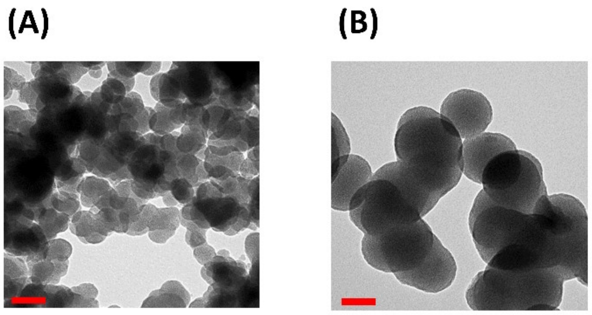

Fig. S3 TEM images of (A) Se$_{10}$-SS-MSN and (B) Se$_{20}$-MSNs. The scale bar is 100 nm.

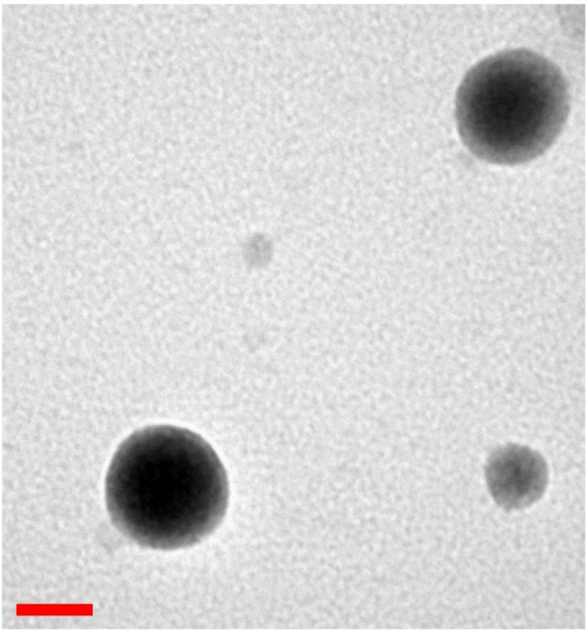

Fig. S4 TEM images of SeNPs. The scale bar is 50 nm.

Table S1 Hydrodynamic size and PDI values of MSN-Se_L, Se-MSNs and SeNP-MSNs in ethanol acquired by DLS

**Samples**	**Hydrodynamic size in ethanol (nm)**	**Pdi**
MSNs	189.2±0.2	0.10
SS-MSNs	299.3±0.3	0.20
MSN-Se_{L10}	177.3±2.5	0.20
MSN-Se_{L20}	192.3±2.2	0.18
Se_{10}-MSNs	279.6±5.0	0.34
Se_{10}-SS-MSNs	854.9±11.6	0.21
Se_{20}-MSNs	1095.5±24.5	0.28
Se_{30}-MSNs	1915.1±67.1	0.29
$SeNP_{25}$-MSNs	316.8±5.3	0.24
$SeNP_{40}$-MSNs	1243.0±5.3	0.21

Table S2 Pore size of as-synthesized MSNs, MSN-SeL$_{10}$, Se$_{30}$-MSNs and SeNP$_{40}$-MSNs determined by BJH method.

**Samples**	MSNs	MSN-Se$_{L10}$	Se$_{30}$-MSNs	SeNP$_{40}$-MSNs
Average pore diameter (nm)	3.27	3.25	2.90	3.06

Table S3 Specific pore volume of as-synthesized MSNs, MSN-SeL$_{10}$, Se$_{30}$-MSNs and SeNP$_{40}$-MSNs determined by BJH method.

**Samples**	MSNs	MSN-Se$_{L10}$	Se$_{30}$-MSNs	SeNP$_{40}$-MSNs
Specific pre volume (cm^3/g)	0.620	0.813	0.562	0.185

Table S4 Specific surface area of as-synthesized MSNs, MSN-SeL$_{10}$, Se$_{30}$-MSNs and SeNP$_{40}$-MSNs determined by BET method.

**Samples**	MSNs	MSN-Se$_{L10}$	Se$_{30}$-MSNs	SeNP$_{40}$-MSNs
Specific surface area (cm^3/g)	745.8	963.5	843.5	228.9

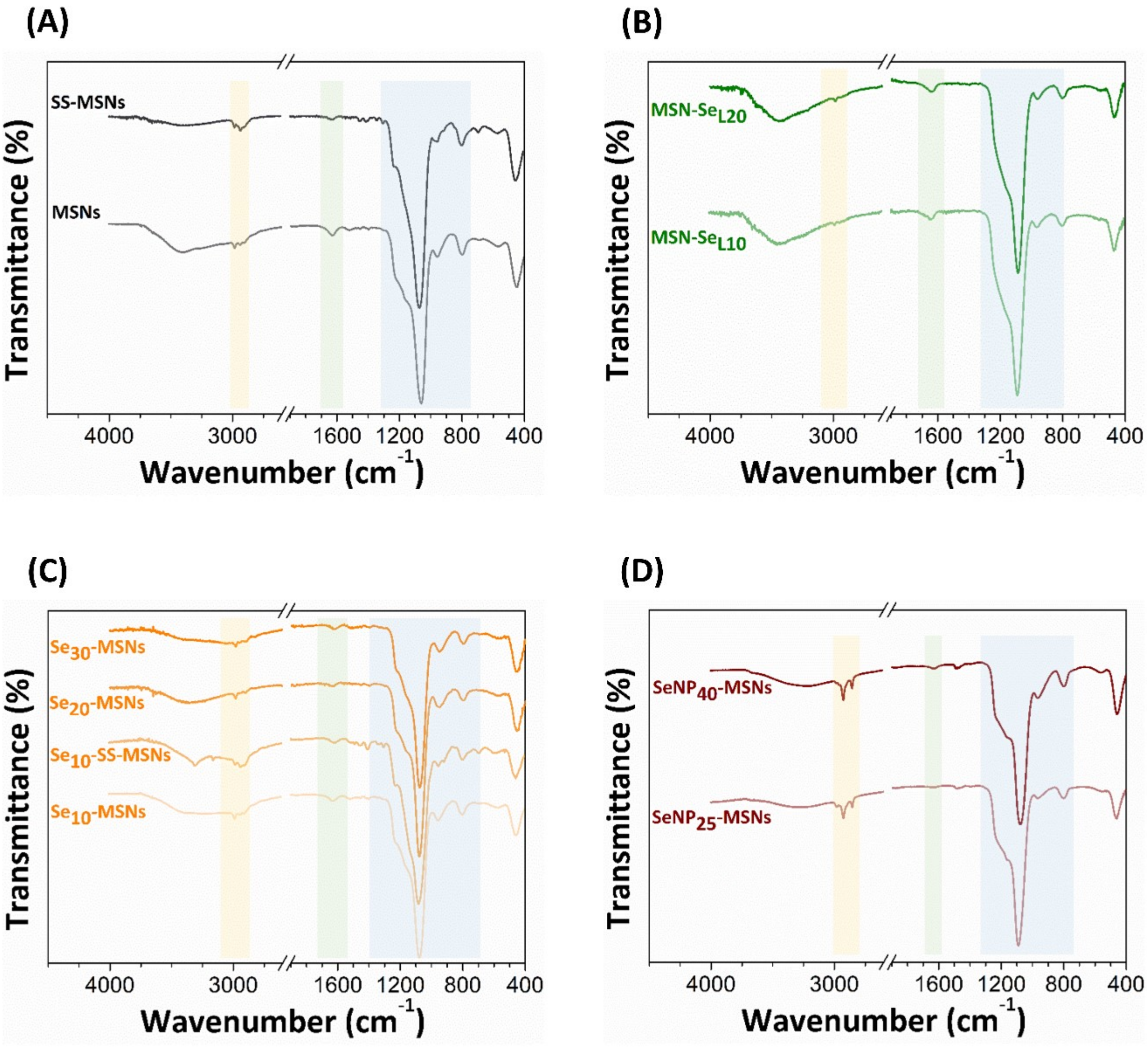

Fig. S5 FTIR spectra of all groups of Se-incorporated MSNs. (A) MSNs and SS-MSNs; (B) MSN-Se_L; (C) Se-MSNs; (D) SeNP-MSNs.

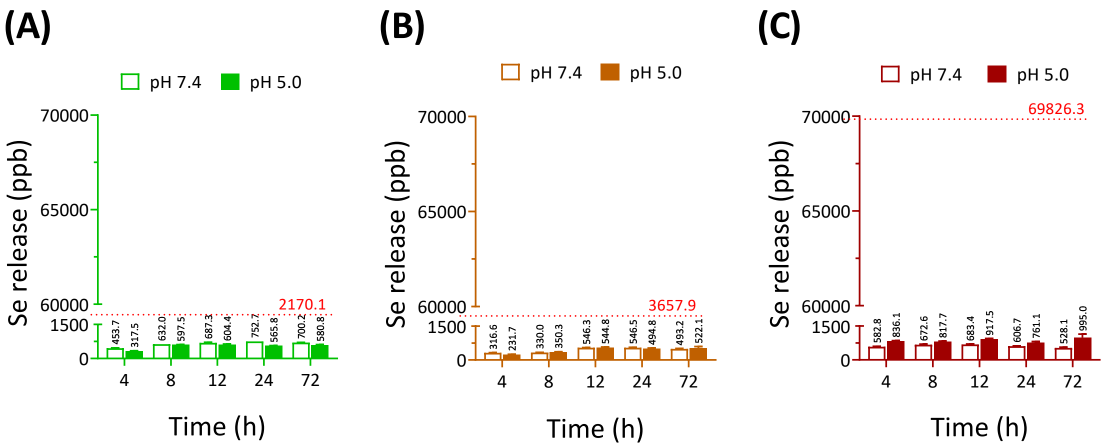

Fig. S6 Amounts of Se (ppb) released from (A) MSN-Se$_{L10}$; (B) Se$_{30}$-MSNs; (C) SeNP$_{40}$-MSNs in different pH conditions (7.4 and 5.0).

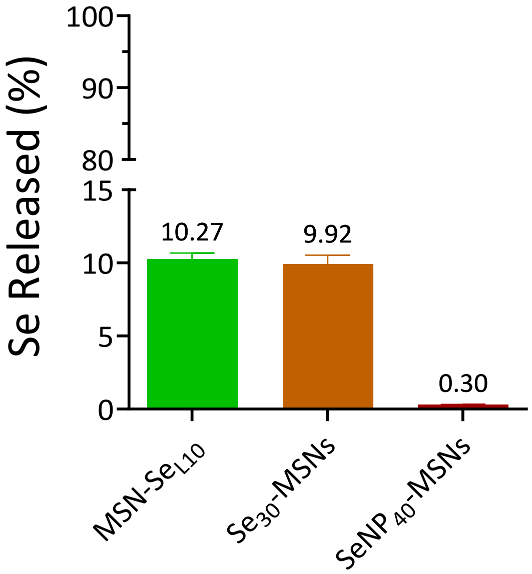

Fig. S7 Biostability of MSN-Se$_{L10}$, Se$_{30}$-MSNs and SeNP$_{40}$-MSNs after immersion in regular cell culture medium (DMEM + 10%FBS) for 14 days.

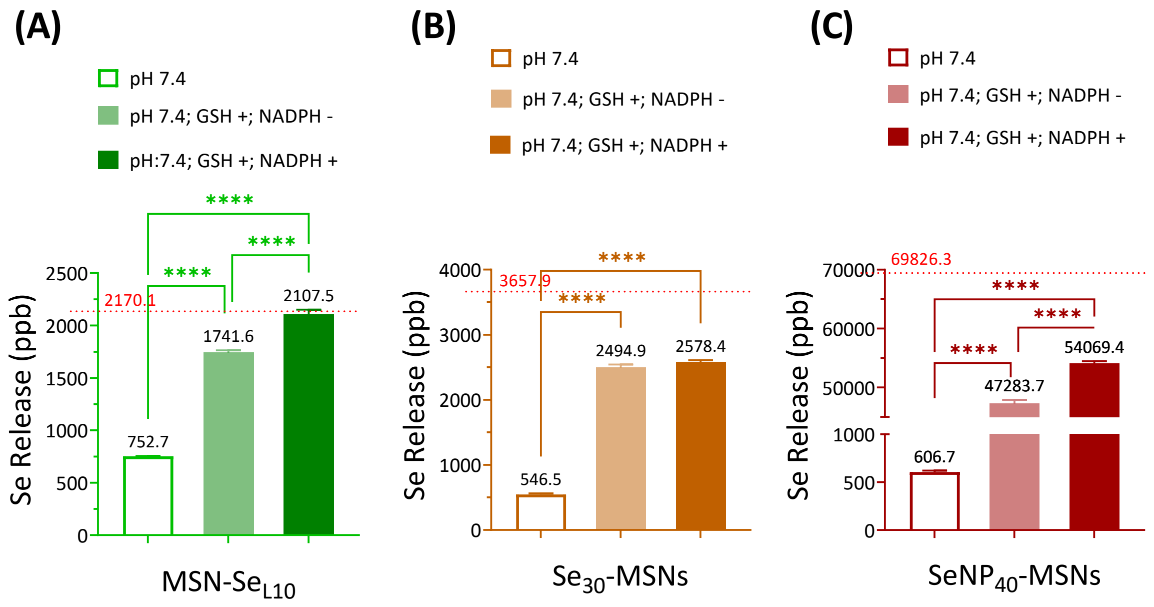

Fig. S8 Amounts of Se (ppb) released from (A) MSN-Se$_{L10}$; (B) Se$_{30}$-MSNs; (C) SeNP$_{40}$-MSNs after 24 h incubation in the absence/presence of GSH (10 mM) and NADPH (1.0 mM) at neutral conditions. n = 3. * represents *p*-values of significant difference compared to the controls. (*: $p < 0.05$; **: $p < 0.005$; ***: $p < 0.001$; ****: $p < 0.0001$).

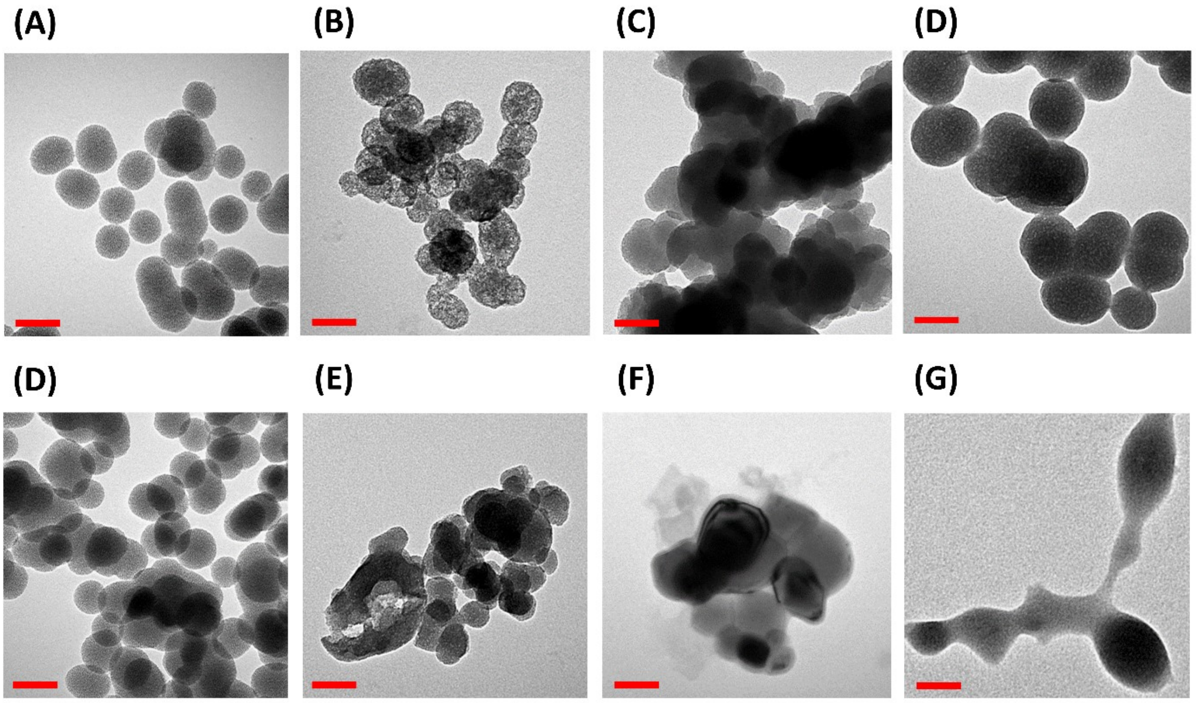

Fig. S9 TEM images of (A) MSNs, (B) MSN-Se$_{L10}$, (C) Se$_{30}$-MSNs, and (D) SeNP$_{40}$-MSNs after 24 h incubation at neutral conditions. TEM images of (D) MSNs, (E) MSN-Se$_{L10}$, (F) Se$_{30}$-MSNs, and (G) SeNP$_{40}$-MSNs after 24 h incubation in the presence of GSH/NADPH at neutral conditions. The scale bars are 100 nm.

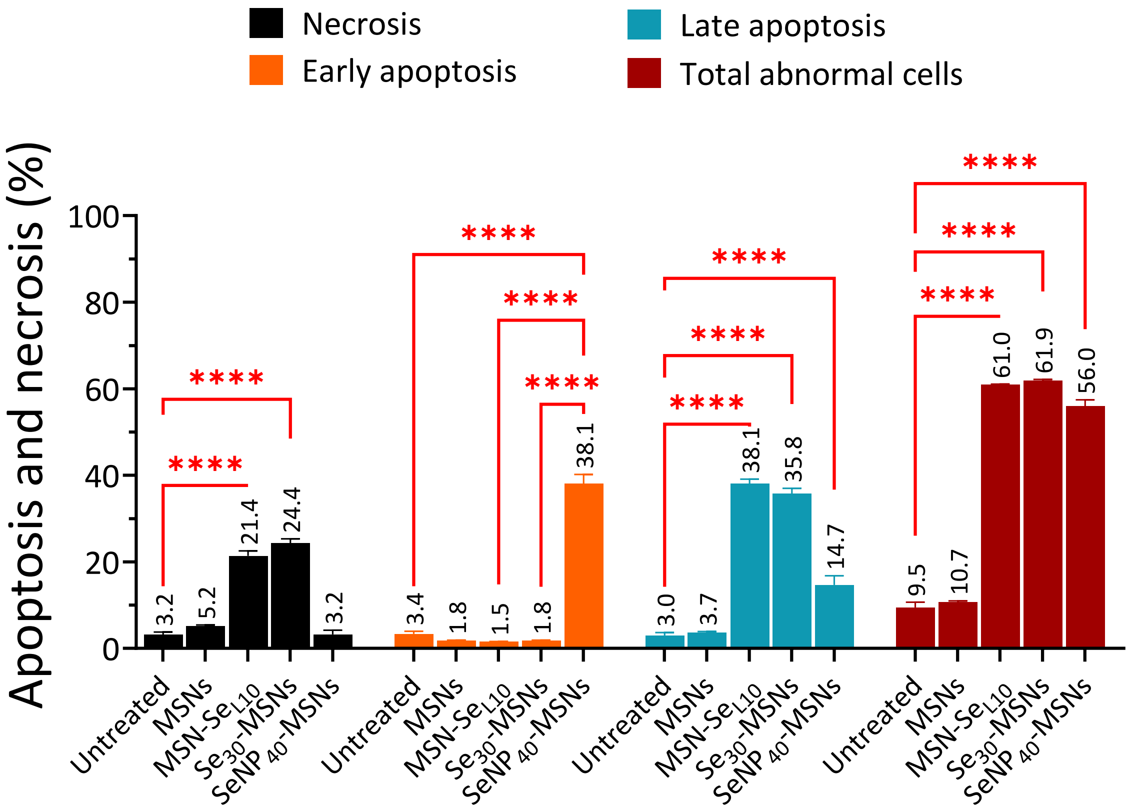

Fig. S10 Early apoptosis, late apoptosis and necrosis of Saos-2 cells upon exposure to different MSNs for 12 h. n = 3. * represents p-values of significant difference compared to the controls. (*: $p < 0.05$; **: $p < 0.005$; ***: $p < 0.001$; ****: $p < 0.0001$).
